# Replaced Right Hepatic Artery Arising From the Celiac Trunk: A Rare and Challenging Anatomical Variant of the Pancreaticoduodenectomy Procedure

**DOI:** 10.7759/cureus.21402

**Published:** 2022-01-19

**Authors:** Arunanshu Behera, Cherring Tandup, Uttam K Thakur, Swapnesh Sahu, Deepak Sutrave

**Affiliations:** 1 General Surgery, Postgraduate Institute of Medical Education and Research, Chandigarh, IND; 2 General and Colorectal Surgery, Postgraduate Institute of Medical Education and Research, Chandigarh, IND; 3 General Surgery, Post Graduate Institute of Medical Education and Research, Chandigarh, IND

**Keywords:** periampullary carcinoma, anatomic variation, pancreaticoduodenectomy, celiac trunk, hepatic artery

## Abstract

Anatomic variation of the hepatic artery is common and often seen in patients with periampullary carcinoma undergoing a pancreatic duodenectomy. Replaced right hepatic artery from the superior mesenteric artery is the most common variant encountered. Here we present a rare case of an unclassified pattern of the variant anatomy of replaced right hepatic artery originating from the celiac trunk along with an accessory left hepatic artery arising from the left gastric artery in a patient with periampullary carcinoma undergoing pancreaticoduodenectomy.

## Introduction

Hepatic arterial anatomical variation is often encountered in patients with pancreatic cancer undergoing pancreaticoduodenectomy (PD). Hepatic arterial anatomical variation is seen in up to 17% of preoperative visceral angiograms [1]. Many classification systems have been described in the literature to categorize the variations [1]. It is important to be aware of variant hepatic arterial anatomy to avoid inadvertent injury or ligation leading to poor outcome, morbidity, and mortality. Here we present a rare case report of an unclassified pattern of arterial anomaly encountered in a patient with periampullary carcinoma undergoing PD.

## Case presentation

A 59-year-old male presented to the surgery clinic with pain in the epigastric region for eight months. He also gave a history of obstructive jaundice for one month, which was gradually progressive. There was an unintentional weight loss of 6 kgs over the past six months and was associated with anorexia. On physical examination, his performance status was found good, Icterus was present. Per abdominal examination was within normal limits. His total bilirubin was 8 mg/dL, with raised alkaline phosphatase 350 U/L (reference range: 40-128 U/L). Cancer antigen (CA) 19.9 was raised to 423 IU/L. 

**Figure 1:**
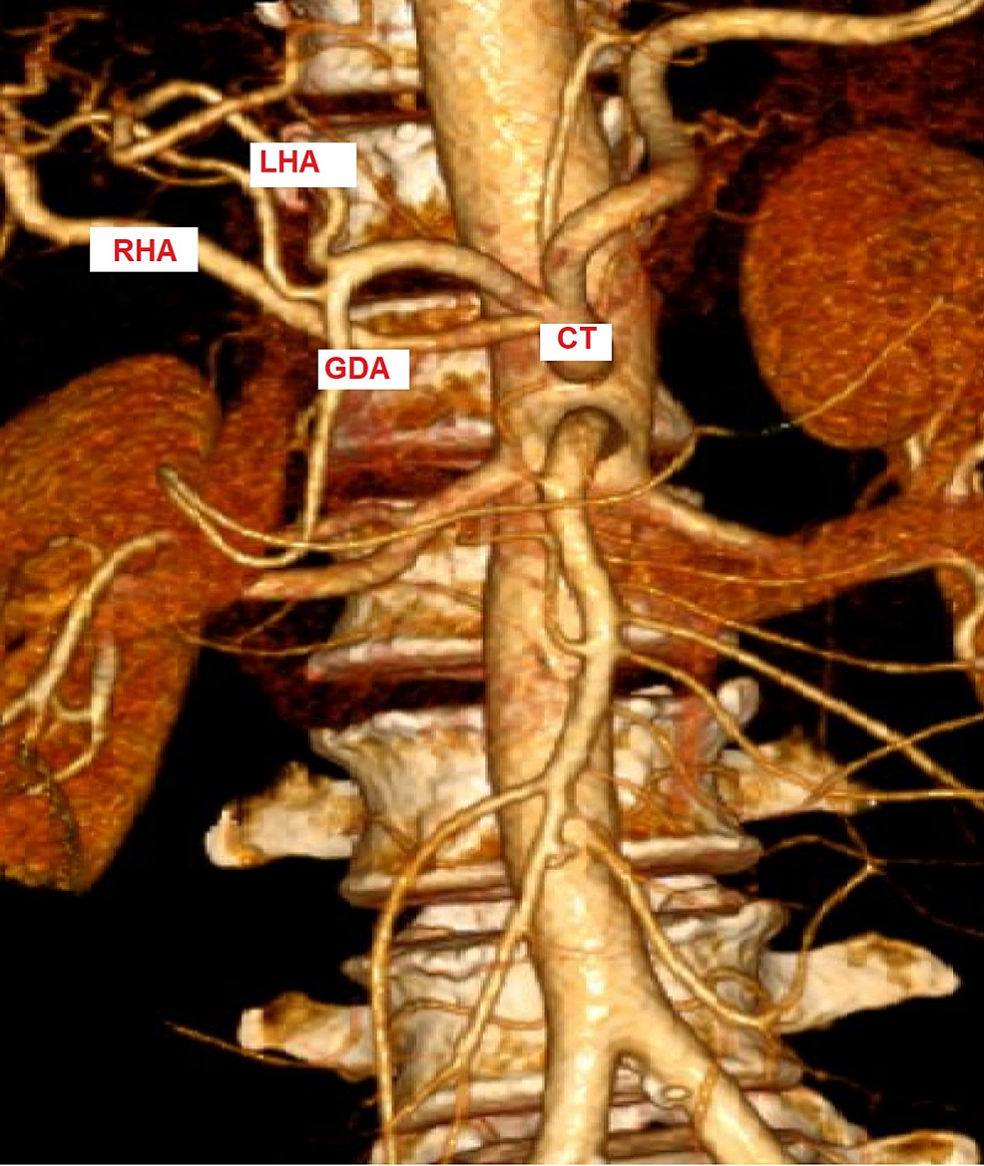
Three-dimensional colored computed tomography showing replaced right hepatic artery arising from the celiac trunk. CT- Celiac Trunk, GDA- Gastro-Duodenal Artery, LHA- Left Hepatic Artery, RHA- Right Hepatic Artery

Ultrasound abdomen showed dilated intrahepatic biliary radicals and common bile duct. Contrast tomography with colored three-dimensional angiography scan showed a 3.4 cm x 2.6 cm hypoenhancing mass in the pancreatic head abutting the right hepatic artery (RHA) for a length of 2.2 cm distal to its origin from the celiac artery. The accessory left hepatic artery (supplying to segments 4a and 4b), was arising from the left gastric artery (Figure 1).

**Figure 2:**
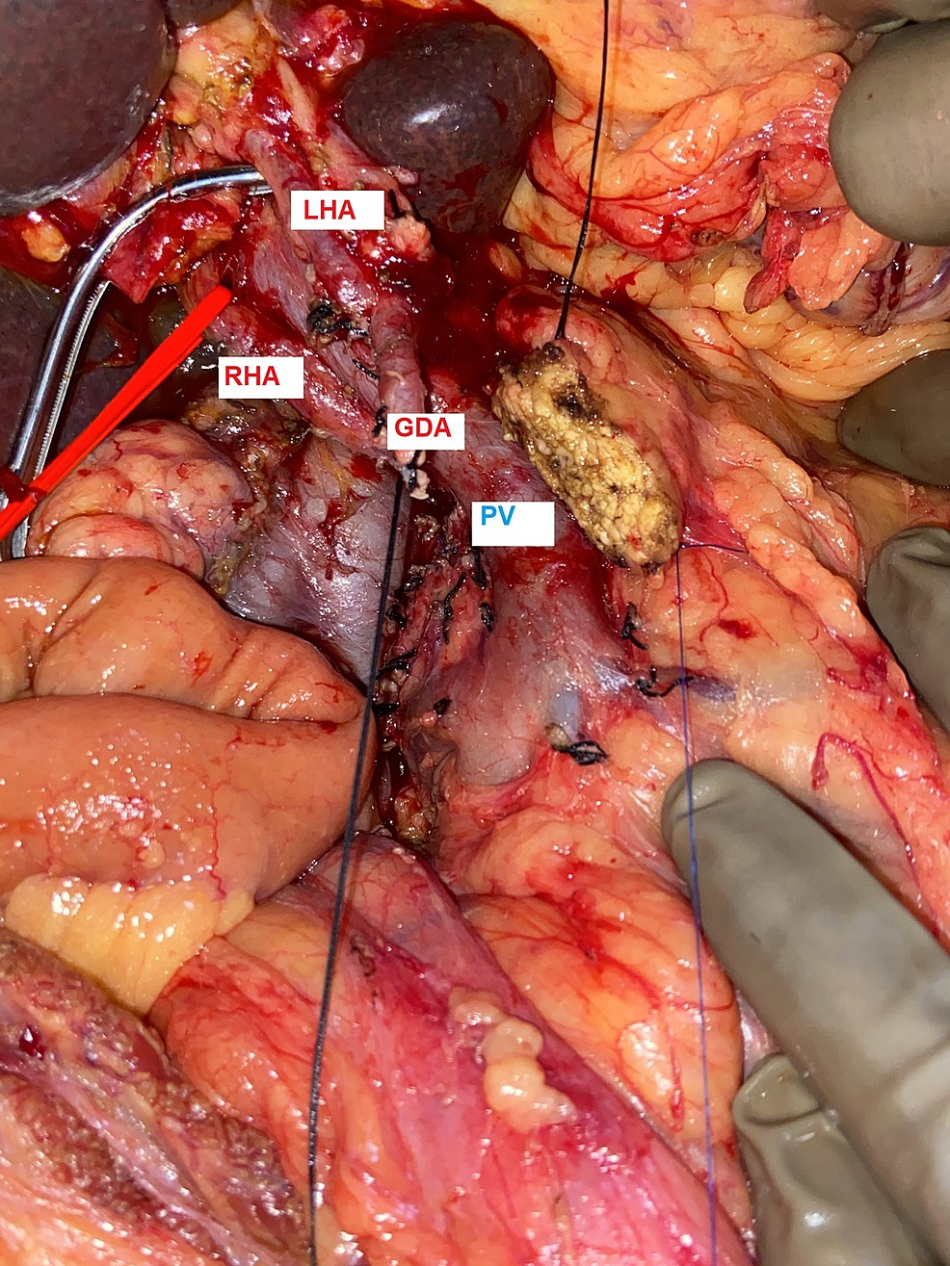
Intraoperative image showing right hepatic artery looped in a red sling, coursing posterior and lateral to the portal vein, along with long gastroduodenal artery stump. GDA- Gastro-Duodenal Artery, LHA- Left Hepatic Artery, RHA- Right Hepatic Artery, PV- Portal Vein

During PD, the intra-operative right hepatic artery variant (Figure 2) was identified, arising directly from the celiac trunk, coursing posterior to the portal vein, and adhering to retro-pancreatic lymph node which was dissected. Accessory left hepatic artery (LHA) was arising from the left gastric artery (LGA). Superior mesenteric artery and vein were normal. Histopathology report showed moderately differentiated adenocarcinoma of the periampullary region with pathological staging pT3pN2pM0. The postoperative course was uneventful and the patient was discharged in a satisfactory condition.

## Discussion

Pancreaticoduodenectomy is the only potentially curative treatment option for periampullary carcinoma. The procedure itself is complicated, arterial anatomical variation makes it further complex and there is a possible risk of inadvertent vascular injury or ligation, which may result in increased blood loss and prolonged operation time. Recognition and management of aberrant RHA are of paramount importance during PD. In 1955, Michels categorized and published hepatic arterial anatomical variations after investigating 200 cadavers and also highlighted the differences between aberrant and accessory hepatic arteries and their importance [2]. The term replaced hepatic artery refers to one having an aberrant or anomalous origin, whereas the accessory artery has an abnormal origin and supplies a section of the liver along with another artery. The index case is an unclassified pattern of variation in which replaced RHA is originating from the celiac trunk and an accessory LHA is seen arising from LGA. 

Iatrogenic cessation of arterial flow to the liver during pancreaticoduodenectomy often leads to short and long-term impacts on the outcome of the procedure. Rapid restoration of arterial flow is indicated whenever it is technically possible and can prevent early fatal complications and late biliary stenosis [3]. A review of literature by Shukla et al. analyzed reports between 1960 to 2008, concerning the influence of hepatic arterial anatomical variation on pancreaticoduodenectomy outcomes [4]. They concluded that awareness of preoperative hepatic arterial anatomical variation would decrease intraoperative vascular injury and thereby associate complications like postoperative hemorrhage and bilioenteric anastomotic leaks. When unexpected hepatic arterial variants are encountered intraoperatively, there is a high chance of vessel injury resulting in hemorrhage, hepatic ischemia, abscess, liver enzyme elevation, and anastomotic biliary leaks. In cases that require aberrant hepatic artery resection during PD due to tumor conditions, surgeons should carefully monitor the postoperative course, while keeping in mind the possible necessity of urgent hepatectomy due to liver necrosis, usually in cases having replaced left hepatic artery [5]. Surgical and oncological outcomes of pancreaticoduodenectomy remain unaffected by the presence of ARHA provided that the anatomy is delineated and appropriately managed [6-8]. It is of the utmost importance to have a preoperative knowledge of variations of arterial anatomy for safe performance of hepatopancreatic-biliary surgery and obtain an optimal result.

## Conclusions

Pancreaticoduodenectomy is a complex and technically demanding procedure and is still associated with high morbidity and mortality rates. The presence of a variation in hepatic arterial anatomy may increase the risk of intraoperative vascular damage. However, they are not considered a contraindication and with good surgical experience, the procedure may be done. Consequently, preoperative identification and interpretive anticipation are necessary to prevent complications.
